# Bibliographical Notices

**Published:** 1849-05

**Authors:** 


### BIBLIOGRAPHICAL NOTICES.

*Obstetrics—the Science and the Art.* By CHARLES D. MEIGS, M. D., Professor of Midwifery and the Diseases of Women and Children, in the Jefferson Medical College, one of the Physicians to the Lying-in Department of the Pennsylvania Hospital, Vice President of the Philadelphia College of Physicians, Member of the American Philosophical Society, of the American Medical Association, etc., etc. With one hundred and twenty illustrations. Philadelphia, Lea & Blanchard, 1849.

The above work will be welcomed most cordially by all who feel an interest in the study of obstetrics. Its author is well known as a practitioner of vast experience, in matters appertaining to midwifery, and the diseases of women and children, and it is truly surprising, how one, who is continually going about doing good in his way, should find time to write so much and so well as does Dr. Meigs. Within the last two or three years, he has published his translation of the work of Colombat de L'Isêre, on the diseases of females, his own work on the same subject, and the present treatise on obstetrics; comprising in all nearly three thousand octavo pages of printed matter. Nor must it be supposed that these works have been hurriedly written, for notwithstanding some defects of style and errors of opinion, it is apparent that the author has reflected at leisure upon most of the subjects of which he treats.

The work before us is divided into four parts ; the first comprising the anatomy of the parts concerned in the acts of reproduction; the second, the physiology of reproduction; the third, the therapeutics and surgery of midwifery ; the fourth, the history of the diseases of the neonatus or young child.

Chapter 1st contains a description of the anatomy of the bony pelvis, and of the soft parts lining its cavity. The necessity for a thorough knowledge of the planes, axes, and diameters of the pelvis, is forcibly pointed out by the author, and for the purpose of illustrating the advantages of a proper inclination of the plane of the superior strait, he has copied some outline figures of the female, from the work of Wigand, which we would advise our readers to consult, in consequence of their novelty, and their practical bearing on the prognosis of labour.

Dr. Meigs regards the separation of the pelvic ligaments as pathological, and not physiological in its character. This opinion, maintained by most obstetricians of the present day, is, we think, correct, though its accuracy has been questioned by some writers on midwifery.

In chapter 2d we have a description of "the mechanism of labour, as it depends upon the relation of the pelvis to the fœtal head." Of the vertex, he admits six positions; and in describing the mechanism of each, he explains the movements of *flexion*, *rotation*, *extension* and *restitution*, with sufficient accuracy for all practical purposes. The movement of *rotation* is attributed to the influence of the inclined planes, of which so much is said in some works on midwifery. To this opinion we cannot assent, since the observations of Dubois, Cazeaux, &c., have proved conclusively to our minds, that rotation in a majority of cases occurs when the head has reached the inferior strait of the pelvis, and is no longer under the directing influence of the inclined planes. Indeed, a fact reported by Cazeaux shows that rotation may be deferred, even until the head has almost passed the posterior commissure of the perineum, in which case the inclined planes could have had no influence whatever.

Chapter 3d contains a description of the form, size, sutures and fontanels of the fœtal head. According to our author, "rigorously speaking, there are but two presentations in midwifery—one of the head, the other of the pelvis." Presentations of the shoulder, arm, face, and forehead, are regarded as deviations from the vertex; those of the feet and knees from the pelvis. The mechanism of the various positions, is minutely described in a subsequent part of the work.

In chapter 4th we have a description of the genitalia, with occasional remarks upon some of the accidents to which these tissues are liable, during pregnancy or parturition.

Chapter 5th will be found to contain an interesting account of the anatomy and physiology of the ovaries, Graafian vesicles, and of the ova contained within them. The author reiterates his opinion, previously expressed in a paper read before the Philosophical Society of Philadelphia, in regard to the vitellary nature of the corpus luteum. This opinion is at variance with the observations of other microscopists, and will require further investigation for the determination of its truth. It is certain, however, that the vitellus and the granular matter of the corpus luteum, differ essentially in function; the former lies within the ovum, and is intended to nourish the fecundated ovum of the mammalia, until it forms an appropriate attachment with the uterus; the latter, placed exterior to the cavity of the ovum, has no such function, because the ovum has escaped from the cavity of the Graafian vesicle. There is no difference between the vitellus of the mammalia and that of the oviparous animal, except that in the former, its presence is necessary only during a short period of embryonic life, while in the latter it is required during the whole term of incubation. Dr. Meigs is of opinion that the true and false corpora lutea only differ in magnitude, not in their essential nature.

In the last paragraph of this chapter, we meet with the following opinion expressed by our author, to which we cannot assent. He says, "It surprises me to see that many able and distinguished writers still cling to the antiquated notions as to the ovaric fecundation, which M. Pouchet has shown to be an impossibility." It has been satisfactorily proved (this is admitted by Dr. Meigs, p. 153) by Bischoff and Barry, that the semen does reach the ovary. If this be true, where is the difficulty? It is not necessary to "believe that the male seed enters into the ovisac, through not the peritoneum only, but through the albuginea and the concentric coats of the ovisac," for these coats of the ovum have been perforated by the organic process of ovulation, and it is through "*the open hilum*," or "*pore*," that the semen comes in contact with the ovum which has not yet escaped from the ovisac. The words marked in italics, are extracted from the work of Dr. Meigs, and it is evident that he believes in the possibility of *ovarian fecundation*, and may be justly charged with clinging "to the antiquated notions as to ovaric fecundation, which Mr. Pouchet has shown to be an impossibility." But our author says, in speaking of ovarian pregnancy, that it cannot be deemed possible, except by supposing that "upon some change of posture of the woman, the further escape of the fecundated ovule might be prevented, the pore being stopped by the pressure of a fold of broad ligament, a loop of intestine or other obstructing cause, and thus the fecundated germ, imprisoned within its cell, might commence its carreer of development, making the ancient follicle which produced it, become its matrix and succedaneous womb up to the time at which it must inevitably burst." In this paragraph we have a clear admission of the possibility, not only of *ovarian fecundation*, but of *ovarian pregnancy*, "*antiquated notions*," to which he is surprised any one should cling. In our opinion, no one can doubt, that ovarian fecundation and ovarian pregnancy are possible, not only in consequence of the positive proof, that the semen may reach the ovary, and be thus brought into contact with the ovule, already divested of its coverings, but also, because many cases of ovarian pregnancy have been reported by those upon whose integrity and judgment we may with confidence rely.

Chapter 6th contains a good account of the function of menstruation. The theory adopted by our author, as to the use of the catamenial flow, is based upon the following assertions. 1. Previous to puberty, the ovaries do not contain any mature ovules, but at the completion of the puberic age, ova are matured in the Graafian vesicles, and continue to ripen, until the female ceases to be susceptible to impregnation, which in this country is about the age of forty-five. 2. The ova ripen periodically; in women once a month, in the lower animals, at stated periods, which vary with the species. 3. During the last days of the development of the ovarian ovule, "the vascular circulation and the nervous intensity are greatly augmented, a state which passes far beyond the boundaries of the stroma itself, and being propagated to the uterus and vagina, renders them the seat of a sanguine affluxion and engorgement. Under such circumstances, the uterus increases its weight, it acquires a redder hue, is more sensitive, and sinks somewhat lower in the pelvis. From such engorgement and affluxion it is delivered by means of the menstrual hemorrhage," &c. &c. Though it is maintained by most of those who have investigated this subject, that no ova are matured previous to puberty, yet Dr. Ritchie asserts positively that such maturation does occur, even during childhood, without any catamenial flow, hence the sanguine congestion of the parts, incident to the maturation of ova, cannot with propriety be considered the cause of the subsequent hemorrhagic discharge. Again, is it true that the ova are matured and discharged at stated periods only? The affirmative side of this question is taken by Pouchet, Raciborski, Bischoff, &c., who affirm that in all cases, in which females dying about the catamenial epoch, have been examined, the evidence of the maturation and expulsion of ovules invariably existed. These assertions are denied by other investigators of the highest authority: thus, Dr. Ashwell states, that he examined the ovaries of three females, during the menstrual flow, in neither of whom was there any evidence of the maturation or expulsion of ova. Dr. Ritchie also maintains, that ovulation occurs, not only during the catamenial flow, but also in the intermenstrual period. These facts completely overthrow this new theory of menstruation. In our opinion, there can be no doubt of the fact that the maturation and expulsion of ova are continually going on; for if this were not so, impregnation would only be probable during the catamenial flow, or a short time after its cessation; a doctrine which, though maintained by eminent authority, is at variance with many well attested observations.

In chapter 7th the pathology and treatment of amenorrhea is discussed in a most masterly manner. We agree with our author in thinking that a failure to menstruate should be regarded rather as a consequence, than a cause of ill health, and therefore, instead of endeavouring to cure these affections by searching among the class of emmenagogues for some specific means of cure, an accurate examination into the state of the general system, and of its component organs, should be instituted; and if disease, either functional or organic, be discovered, the case should be treated upon rational therapeutical principles. Such a plan would be much more certain to correct any irregularity in the catamenial function, than the empirical means more commonly employed for the cure of these diseases.

At page 141, Dr. Meigs lays down, in fifteen propositions, his views in regard to hematosis, the object of which is to prove that the endangium or lining membrane of the blood vessels "contains the force that makes the blood;" for, says he, "soon after the chyle is poured into the cavity of the endangium and becomes exposed to the influence of the oxygen in the lungs, it acquires the character of perfect blood. It is not to the oxygen alone that it is indebted for this morphological development. Contact with the endangium is essential to that development, since the blood loses its physical character as soon as it ceases from that contact." This is a novel doctrine, and without going into a discussion of the physiological explanations of the mode in which chyle and lymph are converted into blood, it may be safely assumed, that the blood can hardly owe its healthy condition to the influence of a membrane, formed, if not from the blood itself, at least simultaneously with it. The germinal membrane—a membrane temporary in its structure and function—absorbs the nutritious material from the yolk, and prepares it for the use of the embryo, by converting it into blood. Whether at this time blood vessels have been formed or not, is a matter of doubt. Carpenter says "that whilst the capillary vessels are being formed by the union of the cavities of these, (the cells of the germinal membrane,) blood discs seem to be developed from the granules or cell germs they contain." Thus it would appear, that the blood vessel is not formed before the blood. This, Dr. Meigs seems to admit at page 179, where he says, "it is a faculty of blood in motion to make a channel or vessel for itself, which vessel is found to be lined with endangium;" and again, at page 180, he remarks that "this heart drives the blood into the embryonal sarcode, wherein the blood makes its own channels, which as they become complete are blood vessels". Independent then of the fact that we possess no positve proof that this endangium exercises so important a part in the function of hematosis, are we not justified in denying the doctrine, that the condition of the blood depends upon that of a membrane, which originally was formed either from the blood itself or co-incidentally with it?

In chapter 8th, under the head of pregnancy, we have an account "of all the changes which take place in the respiratory organs, and in the whole economy of the female, from conception to the end of the puerperal state, as well as a history of the development of the fœtus." Fecundation, is defined the vivification of the ovum; and conception, the fixation of this fecundated ovule either within the uterus, or exterior to its cavity. The time which elapses between these two acts, is a matter of great uncertainty.

In regard to the nature of the decidua, our author does not seem to have made up his own mind, for after describing Hunter's views, and extracting largely from the work of Mr. Coste, he says, "I leave it to the student now to judge for himself as to the nature of the deciduous coat of the womb." This is a very difficult question for a student to decide, and though Dr. Meigs has referred him to some of the best authorities, yet to our surprise he has omitted to mention the names of Reid, Sharpey and Goodsir, an analysis of whose opinions may be found in the last edition of Carpenter's Human Physiology. According to Sharpey and Weber, the deciduous coat is composed of the inner portion of the mucous membrane, which after conception undergoes very considerable changes in its character. This membrane, as observed by Reid, possesses on its free surface a tubular structure, which becomes thickened and increased in vascularity after conception. The orifices of these tubules, in a recently impregnated uterus, are easily detected under the microscope, and seem to be lined with a white epithelium. Between these tubules, Goodsir states that a number of nucleated cells are visible. After conception, all these component parts become much hypertrophied, and at a later period of pregnancy, the decidua is said to separate into two distinct layers, "so different in texture, that they cannot be supposed to have the same origin." On this point the following explanation has been offered by Mr. Goodsir; he says, "from what has now been stated, it appears that the decidua consists of two distinct elements; *the mucous membrane of the uterus*, thickened by a peculiar development, and *a non-vascular cellular substance*, the product of the uterine follicles. The *former* constitutes at a later period the greater part of the *decidua vera;* the latter, the *decidua reflexa.* This view of the constitution of the decidua, clears up the doubts which were entertained, regarding the arrangement of the membranes at the os uteri and entrances of the Fallopian tubes. It is evident that these orifices will be open or closed just as the cellular secretion is more or less plentiful, or in a state of more or less vigorous development." Our only apology for having entered so fully into the detail of these views of the decidua is, that they seem to us to simplify what has hitherto been regarded as a most intricate question.

Passing over the description of the allantois, amnion, &c., we come to that of the placenta. Dr. Meigs informs us that, notwithstanding Hunter's assertion that the placenta consists of a maternal and fœtal portion, he, claiming "the privilege to see with his own eyes," is compelled to adopt the opinion of Velpeau, Seiler, &c., who maintain that the placenta is entirely fœtal in its character. The views of Weber, Coste, Flourens, Bischoff, &c., are referred to; but here again, we find the names of Reid and Goodsir omitted. These observers insist that the placenta consists of two portions—the one maternal, the other fœtal. According to Reid, the blood is sent from the mother through the "curling arteries" of the uterus "into a large sac, formed by the inner coat of the vascular system of the mother, which is intersected in many thousand different directions by the placental tufts projecting into it, like fingers, and pushing its thin wall before them, in the form of sheaths, which closely envelope both the trunk and each individual branch composing these trunks. From this sac the maternal blood is returned by the utero-placental veins." In the above opinion Prof. Goodsir coincides, with some slight modification; the most important of which consists in the discovery, that between the tufts of the chorion and the inner coat of the uterine vascular system which envelopes them, there is found two distinct sets of nucleated cells; the one belonging to the maternal portion of the placenta, is situated between the membrane of the chorial villi and the vascular system of the mother; the other belongs to the fœtal portion of the placenta, and lies between the membrane of the villi and that of the umbilical vessels. The object of the nucleated cells is to absorb material from the blood of the mother, so that it may be conveyed by the umbilical vessels to the body of the fœtus. We would refer our readers to the more recent works on Physiology for a more accurate detail of the opinions of Reid and Goodsir, which constitute, beyond doubt, the prevailing doctrines of the day.

In the description of the fœtal circulation, we find that Dr. Meigs still clings to his theory of cyanosis. Without discussing this question, to which a whole chapter is devoted in a subsequent part of this work, we need only state that our author's theory has not been adopted by other members of the profession, because post-mortem examinations have established the fact, that cyanosis depends upon other causes, and that the simple opening of the foramen ovale is not sufficient of itself to produce this accident, nor would the admixture of the venous or arterial blood, account for the peculiar discolouration of the skin.

The duration of pregnancy, according to Dr. Meigs, may be much protracted beyond the usual period. That this is true to a certain extent, but few medical men will deny; at the same time very few will be found willing to agree with our author in thinking, that gestation may be procrastinated till the twelfth or fourteenth month. Two cases are related by our author for the purpose of substantiating his opinion. The first is that of "Signora N.," reported by Asdrubali. This lady was affected with symptoms of pregnancy about the 1st of March, 1796, at which time she had the misfortune to lose her husband, and "to the grief occasioned by the loss of her spouse, were added great distress and embarrassment connected with the inheritance of his estate, and notwithstanding she early declared the existence of her pregnancy, she was much tormented and baffled by his relatives, who treated her declaration as false." This lady after many sufferings was delivered on the 29th of April, 1797, of twins. In this case the period of gestation was extended to nearly fourteen months. Dr. Meigs says, "I find nothing impossible to believe" in this case. We confess that our confidence in the assertion of the lady, *Signora though she be*, is not so great as our author's, the more especially as it seems, from the above quotation, that the inheritance of a nobleman's estate depended upon the birth of an heir. The motive for deception is so great, that we cannot but reject the validity of a case so extraordinary. The second case is that of Ann Gideon, who supposed herself pregnant in the month of July 1839. On the 20th of November she quickened, and in April was seized with symptoms of labour, the waters were discharged, but the labour pains gradually ceased, and she was not delivered till the 13th of September, 1840. The child was living, and was of medium size. This is a remarkable case, and if true would go very far to prove that the term of utero gestation might be extended beyond twelve months. Its truth, no one could doubt, if the facts of the case were the results of our author's own observations, but they are not, for the patient was not seen by him until her entrance into the hospital, on the 4th day of August, about one month previous to her delivery. To show that Dr. Meigs was not confident of the positive truth of the facts of this case, we make the following extract from his work, he says, "Of course, in relating this case, I do not consider myself responsible for the truth of its statements, further than they are worthy of confidence, in view of the character of the patient herself, and as the facts came under my notice. She had the appearance of perfect candour and sincerity in all that she said about it, and I have no doubt, she thinks her pregnancy began in July, 1839, and ended as I have said on the 13th of September, 1840." When such extraordinary cases occur, we must have them substantiated by undoubted testimony, before we can be induced to believe in their genuine existence, hence we are sure our readers will agree with us in thinking, that the two cases reported by our author by no means establish the fact that pregnancy may be prolonged till the twelfth or fourteenth month. Dr. Atlee of this city has published in the Am. Med. Jour. of Med. Sciences for October, 1846, the history of two apparently well authenticated cases, in which pregnancy was prolonged till the end of the twelfth month, the computation of the term being dated from the time of the cessation of the last catamenial flow. In each of the cases quickening was observable about four and a half months after the disappearance of the catamenia, and in twelve months and a few days the females were delivered of unusually large and healthy children. From the account given by Dr. Atlee, we feel sure that there was no motive for deception, and that the females were convinced of the truth of their own statements, but we cannot understand why the beginning of pregnancy was dated from the cessation of the catamenia. Impregnation may have taken place a few days previous to the subsequent menstrual epoch, (nearly one month later,) and if it did, then the term of utero gestation was prolonged only till the end of the eleventh month—a degree of procrastination within the bounds of probabilities. Neither the period of quickening nor the time which elapsed between this and the subsequent delivery, render our explanation improbable, and we are compelled to deny that these were cases in which pregnancy had really been extended to twelve months. In the determination of this delicate question, we think medical men should be extremely cautious, taking care to ascertain whether the character of the female be reliable, whether there exist any motive for deception, and whether the facts of the case have been carefully observed by an intelligent and capable physician.

In fixing the date of impregnation, Dr. Meigs reckons from the day following the disappearance of the last menstrua, adding to it two hundred and seventy-nine days. This mode of computation is not entirely accurate, because, as Dr. Meigs remarks, it will not apply to the case of those religious Jewesses, who refuse all marital connection until eight days after the disappearance of the discharge. But this is not the only case in which its accuracy would be at fault, since it is undoubtedly true, that impregnation may occur some days before the appearance of the catamenia. In a case reported by Dewees, not only was the female impregnated "within a week" of menstruation, but it was found that fecundation did not, as it usually does, interfere with the next menstrual flow.

Our author after giving us an account of the development of the tissue of the uterus, of its blood vessels and nerves, notices in a concise but satisfactory manner the signs and diseases of pregnancy.

In part third we are brought to the consideration of the more practical portion of our author's obstetrical treatise, and it is so well executed, that we cordially recommend it to our readers as the source from which he can obtain a vast amount of most valuable information. Chapters 9th and 10th contain an admirable account of the physiological and mechanical phenomena of the process of parturition and of the duties of the accoucheur when called to a case of labour.

In chapter 11th, the mechanism of face presentations are well described. Our author regards these as natural, and dwells with much propriety upon the great principle which should guide obstetricians in their management, viz., to take cáre in every position of the face that the chin be brought under the arch of the pubis. Unless this be attended to, whether in instrumental delivery or otherwise, the labour will be rendered exceedingly difficult, terminating almost always in the death of the child.

Chapter 12th contains a good account of breech presentations, which are regarded by Dr. Meigs as natural, though much more fatal to the child than either vertex or face presentations.

In chapter 13th we are brought to the consideration of preternatural labours, or those "that cannot be brought to a safe conclusion by the natural powers of the system." The causes which call for interference in cases of labour are exceedingly numerous, hence we have a great variety of preternatural labours, *some essentially so*, from faulty position or pelvic deformity, *others accidental* from the supervention of hemorrhage, convulsion, exhaustion, &c., &c. Each variety of preternatural labour is separately noticed, and in the main, our author's remarks upon their nature and treatment are exceedingly judicious. One of the best articles is that upon shoulder presentations, a perusal of which will fully repay any one who will study it attentively.

In our author's remarks upon flooding there is much to commend, but in one or two points we are compelled to differ with him in opinion, thus in the management of hemorrhage before birth, after having recommended cold applications, the rupture of the membranes, &c., he says, if "the flow of blood should not be stayed, and the os uteri should still continue to be so rigidly contracted as to make it impossible to turn the child, recourse should be had to the ergot in very small doses, with a view of producing a feeble ergotism or tonic contraction of the womb, not severe enough to injure the child, but yet so strong as to condense the uterine tissue sufficiently to arrest the flow of blood from its vessels." To the use of ergot in this case we must enter our most solemn protest, not upon the ground that it will fail by "the tonic contractions" to arrest the flooding, but because these very "tonic conctractions," besides being exceedingly apt to destroy the child, are unfavourable to the future delivery of the patient, inasmuch as they prevent the dilatation of the os uteri. The plan which we would substitute for the use of ergot, would be the employment of the tampon. Our author objects to the tampon, upon the ground that it serves to convert an open into a concealed hemorrhage, and increases the flow of blood by producing a further separation of the placenta, &c. We do not consider these objections to the use of the tampon as well founded, because the uterus, already distended with the full grown fœtus, will hardly allow any farther flow of blood within its cavity; hence much benefit will finally result from the formation of coagula at the mouths of the uterine vessels. By adopting this plan of treatment, we will avoid the unfavourable influence which the use of ergot rarely fails to exert upon the subsequent progress of labour. The effects of ergot are not so easily controlled as Dr. Meigs seems to think, and we very much fear that in the attempt to induce "feeble ergotism," the most injurious consequences might arise from excessive contraction of the uterine fibres. In post partum hemorrhages, this medicine is most valuable, but in any other form we would regard its employment as extremely injudicious. For the fully expressed views of our author on the effects of ergot, we would refer our readers to chapter 21, which is entirely devoted to the consideration of this subject.

The description of the nature and treatment of the other varieties of hemorrhage, and of the hour-glass contraction, will be found full of interest and highly instructive. We observe that our author refuses to adopt the mode of treatment proposed by Drs. Radford and Simpson in placenta prævia cases. Though this question may at present be regarded as undetermined, yet we are disposed to think, that the new plan of treatment offers greater chances of safety to the mother, than the one usually resorted to by medical men.

In this chapter, convulsions, prolapsus of the cord, fainting, anæmia, hernia, exhaustion, &c., &c., are all judiciously considered.

Chapter 14th contains a full account of pelvic deformities, to which we would refer our readers.

Chapter 15th is devoted to the forceps. The mode of applying these instruments and the cases requiring their employment are well described by Dr. Meigs. In his preference for Davis' forceps we do not agree, but believing, that practice will enable every skilful obstetrician to employ with success most of the varieties of forceps, in use at the present day, we do not regard this difference of opinion as a matter of much importance. Our author in asserting that Dr. Huston of this city employs Siebold's forceps, has unintentionally fallen into an error. The forceps of Dr. H. is a modification of Siebold's, as will seen by reference to the following remarks by Dr. H. in his edition of Churchill's midwifery. "By the kindness of Dr. Mœhring, a former pupil of Siebold, I have before me the instrument of that distinguished professor, made by Windler of Berlin, and which may, therefore, be regarded as accurate. On comparing mine with it I find them to differ as follows: although the instruments are of the same length, the blades of mine are an inch longer than those of Siebold, and the handles from the pivot correspondingly shorter. This brings the lock more completely free from the vulva, when operating at or above the upper strait; at the same time, the shaft, or narrow part of the blade beyond the pivot, constitutes in fact a part of the handle equally effective with the handle proper. The *fenestra* of the blade is nearly double the width of that of the German instrument, and the sweep of the second curve an inch and a quarter greater. The instrument is also four ounces lighter than the Berlin made forceps." Dr. Huston then goes on to mention the advantages of his modification, which appear to us of very great value.

The remainder of part 3d contains a full account of Embryotomy, Hysterotomy, Induction of premature Labour, Inversion of the Uterus, Puerperal Fever, &c. All of these subjects are well treated and require no comment at our hands.

In part 4th, "The history and diseases of the young child," are discussed in three well written chapters, but our notice has already been so much extended that we must pass them over without comment.

In conclusion, we would recommend this treatise on obstetrics to the profession as one worthy the reputation of its distinguished author; and though we have found fault where errors of opinion presented themselves, yet we have done so conscientiously, conceiving it our duty as reviewers, to expose the errors of a work, exactly in proportion to its merit and the authoritative influence of its author.

*A Practical Treatise on the Domestic Management, and most important Diseases of Advanced Life. With an Appendix, &c. &c.* By George E. Day, M. D., Fellow of the Royal College of Physicians, &c. Republished by Lea & Blanchard, Philadelphia.

This last work of Dr. Day may fairly be regarded as a valuable book in many senses of the term. It abounds in practical suggestions, and in matters of detail no less practical, which are evidently the fruit of great personal experience as well as a wide range of careful reading. The clearness and brevity, not to say completeness, with which the author has managed to convey an unusual amount of important information not easily obtainable elsewhere, must render his production at once a welcome and particularly useful addition to our working libraries.

Volume after volume of the newest pattern heaped upon our table attest, in mass, alike the alarming frequency of disease and death in infancy and childhood, and the necessity for making these disasters the subject of especial and laborious study; yet the far more inevitable disorders and fatal terminations of tottering old age, seem to have shared so little in the zeal with which the same earthly pilgrimage is watched and guarded at the outset of its devious career, that extended monographs of the kind, now under notice, are almost entirely unknown in this country and Great Britain, and are scarcely less rare even among the *de omnibus rebus* writers of the European continent.

It is too true that daily observation will ever continue to remind us of the fiat of holy writ, that the days of man's life are but the melancholy three-score years and ten, beyond which no strength can be looked for that is aught else than labour and sorrow. Still, however slightly the feeble art of man may avail us, there is no reason whatever for despair. We need no ghost from the grave to tell us—we need not even this able treatise of Dr. Day, with its invaluable bibliography and index, to convince us that a vast deal may be done under the guidance of an intelligent experience and humanity, to avert the earlier doom which threatens all men in the sere and yellow leaf, and to mitigate the pains and penalties of an existence prolonged beyond the ordinary span.

We would feel bound to offer a cordial welcome, therefore, to this small and unpretending volume, in honour of its title only, and for the sake of the further investigation it will be sure to stimulate around us.

But a close examination of the essay in itself, has fully justified the interest which the nature of its topics, and the reputation of its author as the translator of *Vogel* and *Simon*, and as the able lecturer "*On Chemistry and the Microscope in relation to Practical Medicine*," had already led us to entertain.

There is much throughout his pages that would afford valuable material for comment and quotation in a more elaborate review, than would serve an advantageous purpose here. There are some questions, too, upon which, in our capacity of censors, we might venture to maintain a difference of opinion. We prefer, however, avoiding the delay and embarrassment of the rigorous selection demanded by a necessarily restricted notice, and propose to do all the justice in our power to its merits as a standard work, by confining our present sketch to a very brief analysis, or rather enumeration of its principal contents.

Beginning with a page of addenda, and an admirable bibliography, for a considerable part of which he is indebted to the German monograph of *Canstatt*, whose work he frequently refers to, Dr. Day divides his treatise into thirteen chapters, in each of which distinct and important topics are concisely, but very practically as well as learnedly, discussed.

The first six chapters are occupied respectively with the most important changes occurring in the system in advanced life; the preservation of the health during the same epoch—an excellent chapter;—the medical treatment of advanced life in general—also highly instructive,—climacteric disease, senile marasmus, and the diseases most fatal to persons in advanced life. Then we have chapter VII. devoted to diseases of the respiratory system, and subdivided into seven sections, each treating of one of the usual forms of respiratory disease. Next comes chapter VIII. also subdivided into sections, and treating of the different diseases of the nervous system, these being next in order of gravity and frequency. This is followed by chapter IX., in sections, on the disorders of the digestive tube and its appendages; X., on diseases of the heart; XI., in five very interesting sections, on diseases of the urinary and generative systems; XII., on diseases of the skin, including ulcers of the legs and senile gangrene, in separate sections; and XIII., and the last, on gout and rheumatism. These chapters on the subject proper, are succeeded by an appendix intended to advance the claims to renewed popularity of an old and certainly very respectable remedial measure, rather magniloquently styled the "*thermic treatment*" of lumbago, sciatica, partial paralysis, and other analogous disorders.

This "thermic" method, in which our readers will doubtless recognize the old-fashioned *ironing* or *firing* of stiff-neck memory, has recently been revived into notoriety by Dr. Corrigan of Dublin, and still more developed and improved in application as a potent therapeutic instrument, by Dr. Day. The doctor takes occasion, in his appendix here alluded to, earnestly to advocate its merits, and to justify his commendation by giving the reports of several well marked cases in its favour.

Having thus arrived at the termination of our author's volume, which by, the by, is furnished with a copious index, we bid him God-speed, entirely agreeing with him in the belief expressed in his introduction, that no apology was needed for the appearance of the work, and that though the subject is one of the highest importance, yet it "has been strangely overlooked during the last half century by the physicians of all countries."

Let us hope that, excellent as it appears to be, it will prove but the pioneer to other more extensive enterprises in the same ample and yet comparatively untilled field. To Dr. Day especially, and above all others at the present time, shall we look for the further efficient prosecution of the work. We turn to him advisedly, because, however thankful for what he has already given to us, we cannot help regretting that he had not allowed himself more room throughout his book; and, particularly, that in certain passages he should have contented himself with a tantalizing reference to his other works instead of quoting them at once in full. In the Philadelphia reprint, which is otherwise good enough, this defect is rendered still more embarrassing by the publisher's omission to have the paging of the reference notes so altered, as to make them correspond with the American editions of the works referred to. Where such authorities are within the reach of the student in this country, the different paging has rendered the references in a great degree without avail to him. It is not our business to interfere with the question of international appropriation in matters of pubishing, but we *will* complain, if, when our literary barks change hands and seas, their smaller colours are not equally changed to match them with each other in their pristine uniformity.

---

*Notes on the Medical Application of Electricity.* By William F. Channing, M. D. 12mo. pp. 199. Boston, 1849.

The application of electricity in the cure of disease, has, at various times, claimed a large share of the physician's attention. Like many of the more valuable articles of the materia medica, it has often accomplished excellent results, but failing to do all that enthusiasm demanded, it has from time to time been nearly lost sight of as a therapeutic agent, Latterly, indeed, it seemed to have been resigned pretty much into the hands of empirics, but recently it has been again brought into notice in the course of regular practice, under favourable auspices.

" The object of this work," the author remarks, " is to present, in a reliable form, the results of experience in this revival of electro-medical application, to arrive at general principles, as far as these can be correctly deduced, and to place the materials of practice or investigation in the hands of all who look with hope to the development of this principle, now receiving so general attention abroad, which is so fertile in its applications, so immediate and so safe in its operation."

The subject is treated under various heads, as : *Physiological relations of electricity, forms of medical electricity, means of application, general application to disease, and special application to disease.* It is in reference to the means of application of electricity, and its special application to disease, that the present publication will be found particularly useful. For this purpose, it may be regarded as a convenient manual, in which the practitioner will learn what is known of the utility of the agent in the treatment of particular diseases, and the form of apparatus and best modes of application in the various cases for which it may be employed.

*Anæsthesia, or the employment of Chloroform and Ether in Surgery, Midwifery, &c.* By J. Y. Simpson, M. D., F. R. S. E., Professor of Midwifery in the University of Edinburgh, Physician-Accoucheur to the Queen in Scotland, etc., etc. Lindsay & Blakiston, Philadelphia.

The above work consists of a number of interesting essays on the use of anæsthetic agents, published at different times by the distinguished Professor of Midwifery in the University of Edinburgh. The experience of Dr. Simpson in the use of chloroform and ether, is such, as to command the greatest respect for his opinions in regard to their mode of action, utility, &c. Without concurring entirely with him on these points, we would recommend those wishing to acquaint themselves with the arguments in favour of anæsthesia, to peruse the several communications of the Edinburgh Professor, contained in the above volume. The work is well printed by the publishers, Messrs. Lindsay & Blakiston, of this city.

---

*A Dictionary of Dental Science, Biography, Bibliography, and Medical Terminology.* By Chapin A. Harris, M. D., D. D. S., Professor of the Principles and Practice of Dental Surgery in the Baltimore College, Author of Principles and Practice ot Dental Surgery, &c., &c. Philadelphia. Lindsay & Blakiston. 1849. pp. 780.

The work before us fills a void that has long existed to those engaged in the practice of dental surgery, and the task could not have devolved upon one better calculated to perform it than upon the author of the imposing volume whose title heads this notice This branch of surgery is extending so rapidly, and rising to such importance in the community, that a cyclopædia embracing satisfactory definitions of its technicalities, and a compendium of necessary, important, and curious collateral information, seemed indispensable. The large and valuable medical and surgical dictionaries of the present day, although invaluable to the student and practitioner of general medical science, contain little that is useful to the practical dentist. To supply this want, therefore, the work before us was undertaken. In it are contained accurate, though necessarily condensed, accounts of the physiological and various pathological conditions of the teeth, and the operations necessary for their cure, together with full descriptions of the instruments and materials needed in them. Interspersed with these are biographical notices of eminent dentists, (written with a truthfulness that we can vouch for in several instances,) and bibliographical notices of their works. Medical, Surgical and Anatomical Terminologies are also introduced through the volume, thereby greatly increasing its value. In fact, the work is an enduring monument to the patience and industry of its author, and one that we should consider indispensable to the practitioner of dental surgery, and would gladly see in the library of every physician and surgeon.

